# A Novel Venom-Derived Peptide for Brachytherapy of Glioblastoma: Preclinical Studies in Mice

**DOI:** 10.3390/molecules23112918

**Published:** 2018-11-08

**Authors:** Steve Swenson, Radu O. Minea, Cao Duc Tuan, Thu-Zan Thein, Thomas C. Chen, Francis S. Markland

**Affiliations:** 1 Department of Biochemistry and Molecular Medicine, University of Southern California, Los Angeles, CA 90089, USA; sswenson@usc.edu; 2 Department of Neurological Surgery, University of Southern California, Los Angeles, CA 90089, USA; minea@usc.edu (R.O.M.); tthein@usc.edu (T.-Z.T.); tchen68670@gmail.com (T.C.C.); 3 Faculty of Pharmacy, Haiphong University of Medicine and Pharmacy, Haiphong, Vietnam; caoductuan11@gmail.com

**Keywords:** disintegrin, glioblastoma, integrin, brachytherapy, radioiodine, vicrostatin, cancer therapy

## Abstract

We developed a bacterial expression system to produce a recombinant disintegrin, vicrostatin (VCN), whose structure is based on a natural disintegrin isolated from southern copperhead snake venom. Our goal is to develop VCN for potential clinical translation as an anti-cancer agent. VCN is a peptide of 69 amino acids with a single tyrosine residue. We have employed VCN as integrin-targeted radionuclide therapy (brachytherapy) for treatment of glioblastoma (GBM, glioma). GBM is a deadly brain cancer that doesn’t discriminate between sexes and knows no age limit. We established that the tyrosine residue in VCN can be radioiodinated with full retention of bioactivity. ^131^I-VCN was utilized for integrin-targeted radionuclide therapy using mouse models of glioma. The combination of radioiodinated VCN plus temozolomide (a DNA alkylating agent) significantly prolonged survival of glioma-bearing mice. We also obtained similar results using an immunocompetent mouse model and a murine glioma cell line. In summary, as demonstrated in studies reported here we have shown that VCN as targeted radionuclide therapy for GBM has significant translational potential for therapy of this deadly disease.

## 1. Introduction

Glioblastoma multiforme (GBM) affects males and females and knows no age barrier; it accounts for more than 50% of all human primary brain tumors. Despite advances in surgery, chemotherapy, and radiation therapy GBM remains a highly invasive cancer, and the average life span is only 12 to 15 months from time of diagnosis. Increasing incidence of GBM is one of the prime factors driving the development of new therapeutic strategies. GBM is considered the most aggressive form of brain cancer; it grows rapidly and spreads into the normal brain. The National Cancer Institute (NCI) estimates that 23,880 adults will be diagnosed with brain and other nervous system forms of cancer in 2018. The NCI also estimates that in 2018, 16,830 of these diagnoses will result in death [1]. The mainstay of treatment for GBM is surgery, followed by radiation and chemotherapy. The main objective of surgery is to remove as much of the tumor as possible without injuring the surrounding normal brain tissue needed for neurological function [2]. Surgical debulking can have multiple positive effects on patient survival including relieving the tumor mass effect, decreasing intracranial pressure, reducing associated brain edema, and lowering steroid dependence, all of which can lead to increased overall patient survival and a higher quality of life [2]. However, GBM is surrounded by a zone of migrating, infiltrating tumor cells that invade surrounding tissues, which uniformly makes the complete removal of the tumor very challenging.

Temozolomide is a leading molecule for the treatment of newly diagnosed GBM [3]. The gold standard of therapy for newly diagnosed patients with primary GBM was first introduced more than a decade ago and is called the Stupp protocol [4]. This consists of a regimen of conformal radiotherapy (dosed fractionated at about 2 Gy/day for a total cumulative dose of 60 Gy) administered concurrently with temozolomide dosed at 75 mg/m^2^ for 42 consecutive days, followed by multiple cycles of adjuvant temozolomide administered for 5 days on and 23 days off while dose is escalated up to 150 mg/m^2^. Although it represented an important therapeutic advancement at the time of its implementation, the survival gains brought by the Stupp protocol are rather modest because resistance to temozolomide emerges rapidly in GBM and recurrence is typical for these patients. In addition to temozolomide there are only limited therapies available for the treatment of recurrent GBM with very limited survival gains. For recurrent GBM surgical management is a significantly more difficult problem and standard procedures are not fully developed at present. However, the current pipeline of experimental therapeutics for recurrent GBM is strong and includes biologics, small molecules, devices, innovative surgical strategies, and immunotherapy including several approaches in phase I and phase II clinical trials. A large group of immunotherapeutic approaches are currently undergoing clinical study for safety and efficacy in the treatment of newly diagnosed or recurrent high-grade gliomas. Further, combination therapies using immunotherapy and other forms of therapy are being explored [5]. Some of these new forms of immunotherapy have shown promise, but a significant time delay will be involved for further testing and clinical trials before their incorporation into GBM standard therapy [6]. Gaining better understanding of the disease will improve what can be achieved through an increase in understanding of molecular mechanisms and the development of clinical trials leading to more promising and effective therapeutic options. In aggregate, recurrent GBM poses a difficult challenge, as the desire to provide significant lesion removal needs to be balanced by the need to preserve neurological function and to retain a good quality of life [7].

As indicated, due to its highly infiltrative nature, GBM is a complicated disease and thus its treatment is a challenge, forcing researchers into investigating novel diagnostic strategies and unique chemotherapeutic approaches. The tumor microenvironment plays a large role in cancer progression and its pharmacological manipulation can repress progression in the recurrence setting. Central to the ability of GBM cells to invade the surrounding brain distant from the tumor is a class of glycoproteins called integrins. Integrins are heterodimeric cell surface adhesion receptors (containing α and β chains) operating at the interface between the extracellular matrix and cytoskeletal apparatus [8,9,10]. Integrins link cytoskeletal proteins to the extracellular matrix and are involved in bi-directional signaling to alter cellular functions. Among these interactions are the adhesion of both endothelial and glioma cells to extracellular matrix proteins and other interactions that are integral to tumor growth, metastasis and angiogenesis. Integrins allow cells to mechanically sense their environment [11] by integrating multiple signaling pathways initiated by extracellular cues with the cellular locomotor apparatus. Integrins exhibit structural diversity and undergo conformational changes that are central to the regulation of their receptor function. They exist in three major conformational states: an inactive or low affinity state, a primed or activated high affinity state, and a ligand bound or occupied state [8]. Activated integrins are not usually present in quiescent tissues, but some, such as the RGD-dependent ones which recognize the Arg-Gly-Asp (RGD) sequence present in key extracellular matrix proteins [12], play an important role in neoplastic processes including glioma progression [13], invasion (via invadosome formation) [14,15], angiogenesis [16,17] and dissemination. This subject was effectively reviewed in [18].

Disintegrins represent evolution’s integrin targeting “invention”. Disintegrins are a class of disulfide-rich peptides, originally isolated from snake venom [19,20,21]. These peptides hold a significant translational potential with desirable pharmacological attributes based on their high-affinity/high-specificity interaction with tumor integrins. The integrin-binding activity of disintegrins depends on the appropriate pairing of several cysteine residues responsible for the disintegrin fold, a mobile 11-amino acid loop protruding from the polypeptide core displaying a tripeptide motif, RGD in many disintegrins [22,23]. It is important to emphasize that disintegrins bind only to the activated conformations of integrins on motile cells such as cancer cells and angiogenic endothelial cells [8,21], making them attractive vehicles to specifically target cancerous tissues. We and others demonstrated that disintegrins are well tolerated and can be infused without toxicity or detrimental effect on blood pressure, body temperature, or other physiological parameters [24,25,26]. Subsequently, our laboratory designed a sequence-engineered RGD-disintegrin, vicrostatin (VCN), that can be reliably produced in large quantity (~200 mg/L purified VCN from bacterial cell lysate) in a robust recombinant bacterial system [27,28]. Recombinant VCN retains the binding properties of the natural disintegrin from which it originated, the homodimer contortrostatin (CN) isolated from southern copperhead snake venom, while showing improved binding affinity, compared to native CN, for an important integrin in angiogenesis, integrin α5β1.

The characteristic expression of very high densities of active RGD-dependent integrins by GBM (expressed both by its florid neovasculature and by the tumor cells themselves) creates a unique opportunity for precision delivery of minute doses of beta-emitting radionuclides (such as ^131^I) into the tumor microenvironment via highly targeted polypeptides such as VCN. In the present report we describe our findings with the use of radioiodinated-VCN as a form of integrin-targeted radionuclide therapy (brachytherapy) in combination with temozolomide in murine models of human GBM.

## 2. Results

### 2.1. Structures of VCN and CN

**Figure 1:**
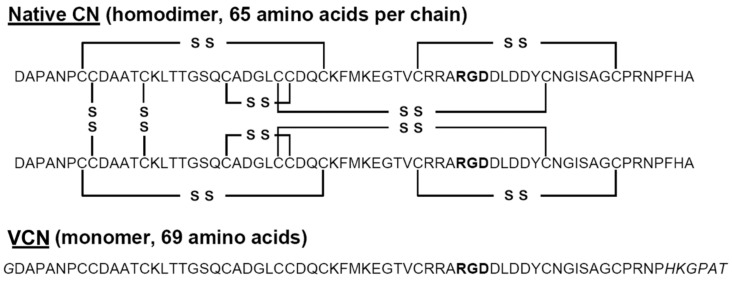
Sequence alignment of native contortrostatin (CN) and recombinant vicrostatin (VCN). The recombinant disintegrin carries a short C-terminal amino acid sequence grafted from another snake venom disintegrin (echistatin) which improves VCN’s affinity toward α5β1 integrin and also causes the recombinant disintegrin to fold as a monomer. A unique tyrosine residue is available for radioiodination in the C-terminal half of VCN (residue 51). The RGD sequence is bold type.

The comparative amino acid sequences of VCN and its predecessor CN are shown (Figure 1). The extension at the COOH-terminus of VCN is clearly visible and was purposefully engineered into the VCN structure [28] to improve binding affinity to integrin α5β1, which it accomplished by about 13-fold as compared to the affinity of CN for this integrin. Further, this alteration at the COOH-terminus had very minimal effect on the affinities of VCN for integrins αvβ3 and αvβ5. Additionally, the tyrosine is shown at residue 51 in VCN, which is the site of radioiodination of the peptide.

### 2.2. Evaluation of Tumor Specific Brain Uptake of Radioiodinated VCN

To identify the ability to iodinate and produce an effective iodination stoichiometry we examined several methods of iodination. Ultimately, using either radioinert or radioactive iodine, we used the chloramine-T procedure [29] for VCN iodination. We confirmed retention of biological activity of iodinated VCN by its ability to inhibit platelet aggregation with identical activity to that on non-iodinated VCN. Additionally, the labeled material was analyzed by mass spectrometry and we observed a ~70:30 mixture of mono-iodinated to di-iodinated material in the reaction which yielded VCN with full activity. Allowing the reaction to yield more of the di-iodinated material resulted in reduced biological activity, whereas attempting to produce only mono-iodinated material resulted in a high level of un-iodinated species which defeats the ability to deliver radioactivity to the tumor.

### 2.3. Confirmation of Iodinated VCN Binding to Glioma Cells and Tumors

**Figure 2:**
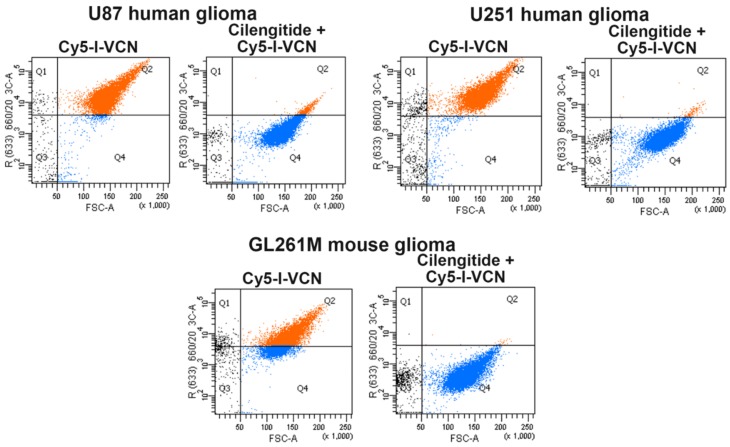
Iodinated VCN avidly binds to glioma cell lines. A FACS analysis was conducted with Cy5-labeled iodinated VCN (Cy5-I-VCN). TMZ-sensitive human glioma cell lines (U87 and U251) or TMZ-resistant mouse glioma cells (GL261M) were incubated with Cy5-I-VCN in the presence or absence of molar excess (100-fold) of a small cyclic RGD peptide competitor (cilengitide).

All glioma cells used in the study were analyzed by FACS for integrin expression levels (i.e., αv and α5 integrin subclasses) and found to overexpress active species of these integrin members (data not shown). VCN labeled with radioinert iodine (i.e., I-VCN) was further labeled with Cy5-NHS to assess the ability of Cy5-I-VCN to bind to glioma cells. This FACS analysis revealed avid binding by Cy5-I-VCN to all glioma cell lines used in our studies. To confirm receptor specificity, the binding of Cy5-I-VCN was inhibited when the cells were preincubated with 100-fold molar excess of cilengitide, a methylated cyclic RGDfV peptide targeted to αvβ3 and αvβ5 integrins [30] (developed by Merck KGaA, Darmstadt, Germany), before the addition of fluorescent disintegrin (Figure 2).

Furthermore, using ^125^I-VCN, we evaluated the ability of the radioiodinated disintegrin to be delivered intravenously (IV) and be retained in the brain and or blood vessels supplying the brain after 18 hours of circulation time. We found that a significant portion of the IV delivered material is found in the brain and associated vasculature in U87 glioma-bearing animals while non-tumor bearing animals display minimal uptake in the brain (Table 1). In the control group no counts above background were observed of the 100 μCi (2.2 × 10^8^ counts per minute, cpm) injected, while in the tumor bearing animals an average of 1.4% (3.1 × 10^6^ cpm) of the injected counts were concentrated in the brain.

### 2.4. Determination of Efficacy of ^131^I-VCN in Limiting Tumor Progression in Xenograft Models of GBM

**Figure 3:**
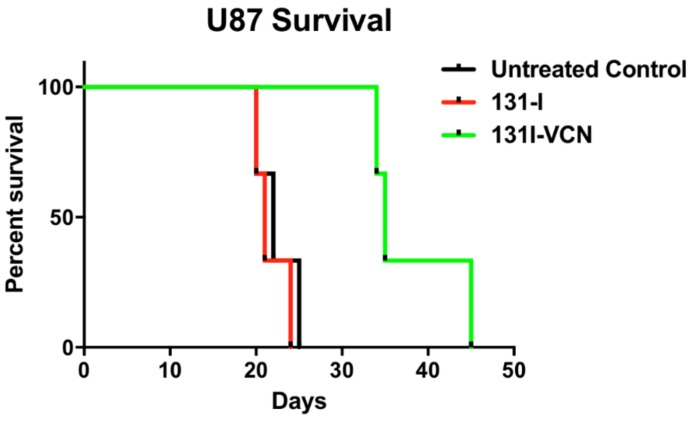
Survival data following treatment with ^131^I-VCN in the U87 xenograft model (5 mice per group). Treatment with ^131^I-VCN extends survival of treated animals by more than 13 days (60% extension). Animals treated with ^131^I-VCN maintained generally a good appearance and stable weight until a few days before succumbing to the disease. There is a significant therapeutic effect of ^131^I-VCN (*p* value 0.0339) as compared to the control.

We evaluated the efficacy ^131^I-VCN in prolonging the progression-free survival of study animals in two distinct xenograft models of glioma that are devoid of O-6-methylguanine methyltransferase (*MGMT*) expression (i.e., U87 and U251) and therefore sensitive to TMZ. The U87 mice in the control groups, began dying on day 20 and all mice in the control and ^131^I alone groups were dead by day 25. The mice treated with ^131^I-VCN displayed no adverse effects of treatment and started to die on day 34 with all mice dead by day 44. The median survival for the controls, untreated and ^131^I alone, were 22 and 21 days, respectively. While for ^131^I-VCN, a median survival of 35 days was observed (Figure 3).

**Figure 4:**
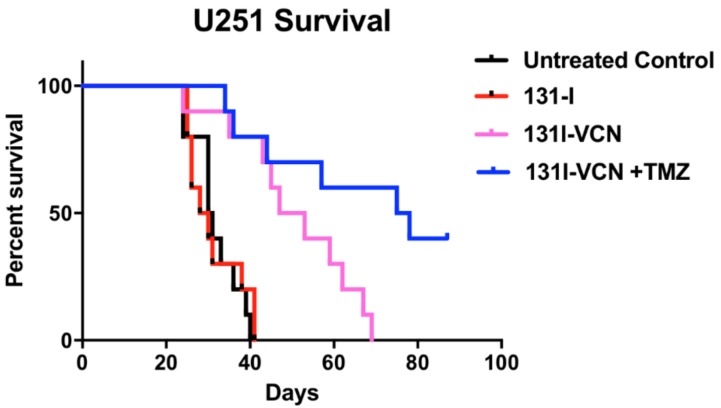
Survival data following treatment with ^131^I-VCN with or without temozolomide (TMZ) in the TMZ-sensitive U251 xenograft model. Treatment of tumor bearing animals (10 mice per group) with the combination of ^131^I-VCN plus TMZ significantly enhances their survival over the animals treated with ^131^I-VCN alone with a median survival in the combination group greater than 75 days. Furthermore, several mice from the combination group appeared tumor free at the conclusion of the experiment. Both the ^131^I-VCN (*p*-value 0.0022) and ^131^I-VCN + TMZ (*p*-value 0.0003) groups show a significant therapeutic advantage over the control while ^131^I shows no significant therapeutic effect.

Next, we examined the efficacy of ^131^I-VCN in the U251 glioma model when delivered with the DNA alkylating agent, temozolomide (TMZ). TMZ functions by alkylating/methylating DNA nucleotide bases with >90% of lesions occurring at many different positions in all bases (i.e., the non O6-lesions) and a minority (<10%) of lesions occurring at the O-6 position of guanine residues (i.e., the O6-lesions). When the promoters of *MGMT* are methylated, the resulting O6 lesions will trigger the death of the tumor cells since the transcriptional silencing of the *MGMT* gene prevents the synthesis of the enzyme that repairs DNA damage inflicted by TMZ. In this study, the U251 tumors were implanted as described for the U87 model and tumor growth was confirmed 7 days post implantation. Animals in the control groups had an average median survival of 30 days. The ^131^I-VCN and ^131^I-VCN plus TMZ didn’t lose weight until late into the study and slowly began dying on day 42 with the final survivor in the ^131^I-VCN group surviving 69 days. Three of the 10 ^131^I-VCN plus TMZ mice died prior to day 57 with another two dying by day 78 and the remainder surviving beyond 90 days (Figure 4). One of the long surviving mice began displaying signs of neurological impairment and re-growth of the tumor was confirmed by optical imaging. Once confirmed, this mouse had the tumor harvested, mechanically dissociated and cells placed in culture, expanded and stored for future analysis.

### 2.5. Comparison of Efficacy of ^131^I-VCN in a Syngeneic Model of TMZ-Resistance Following a Schedule of Administration Designed to Mimic the Stupp Protocol

**Figure 5:**
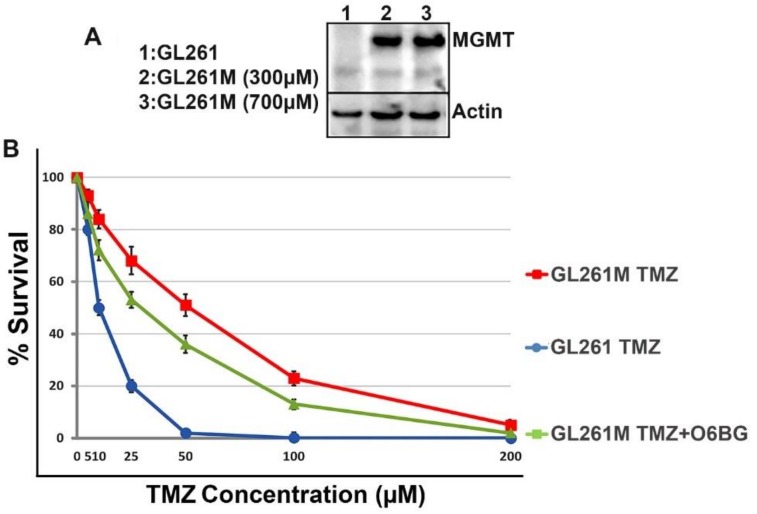
GL261M cells express functional mouse MGMT. (**Panel A**) Lysates were prepared from *MGMT*-transfected cells (GL261M) selected in the presence of increasing concentrations of TMZ and analyzed by Western blotting for MGMT expression. (**Panel B**) The functional activity of MGMT was further validated in a colony survival assay which shows that GL261M cells are resistant to TMZ but are re-sensitized to the drug in the presence of 10 μM of O6-benzylguanine (O6BG).

In this study, we compared the efficacy of ^131^I-VCN plus TMZ against the current standard of care {i.e., external beam radiation therapy (EBRT) plus TMZ} for glioma. We first derived a TMZ-resistant population of murine glioma cells after transfecting the GL261 cells with a plasmid construct carrying *MGMT* placed under the control of a medium-strength mouse promoter. To select highly resistant TMZ cells, the transfected cells were further incubated with increasing concentrations of TMZ (up to 700 μM). The cells derived after this procedure (i.e., GL261M) were further analyzed by Western blotting for MGMT expression and confirmed that the GL261M cells are indeed producing MGMT. Furthermore, we validated the functionality of this artificially expressed MGMT enzyme in a colony forming assay (CFA) by seeding wildtype GL261 and GL261M at very low densities (50 cells/cm^2^) and incubating them in the presence of a range of TMZ concentrations with or without O6-benzylguanine (O6BG), a potent MGMT inhibitor (Figure 5).

**Figure 6:**
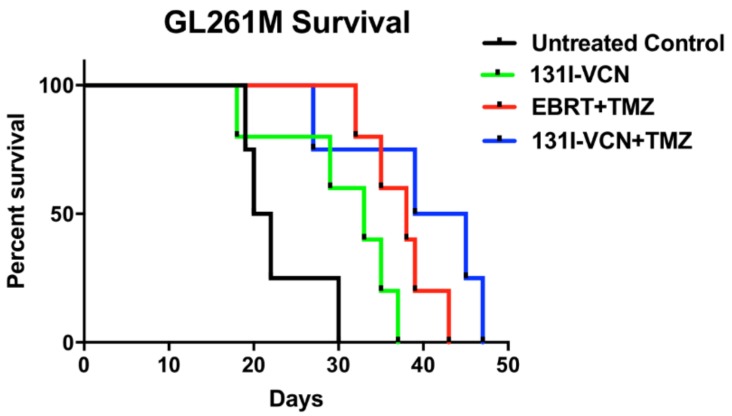
Survival data following a Stupp protocol-like schedule in a TMZ-resistant GL261M syngeneic model. Animals (5 mice per group) in this model were treated with either external beam radiotherapy (EBRT) dosed at 2 Gy/day plus TMZ dosed at 25 mg/kg/day both administered for five consecutive days or with 2 doses of 100 μCi ^131^I-VCN (administered 3 days apart) either alone or in combination with TMZ dosed at 25 mg/kg/day for five consecutive days. In this glioma model of TMZ-resistance, the ^131^I-VCN/TMZ treatment combination shows better efficacy compared to the standard of care EBRT/TMZ combination. There is a significant therapeutic effect between EBRT + TMZ (*p*-value 0.0047) and ^131^I-VCN + TMZ (*p*-value 0.0328) when compared to the control.

Once the TMZ-resistant model was validated in vitro, we implanted syngeneic immunocompetent mice with GL261M cells and allowed tumors to grow and become well established for 12 days after implantation. These tumor-bearing mice were further randomized into four groups and treatment was initiated 12 days post tumor implantation. The untreated control and ^131^I-VCN groups began to show severe signs of tumor growth (lethargy, weight loss and poor body condition score) by day 15 and began to die on day 19 post implantation with all animals being sacrificed by day 30 in the control group (median survival 21 days) and day 37 for the ^131^I-VCN (median survival 33 days). The combination of EBRT with TMZ, which was designed to mimic the Stupp protocol [4], demonstrated much better survival compared to the controls (i.e., the first animal succumbing to the tumor at day 32), and with a mean survival of 38 days. The combination of ^131^I-VCN with TMZ, overall, performed the best with the first animal succumbing on day 27, but with a median survival of 42 days for this group (Figure 6).

## 3. Discussion

Intratumor implantation of radioactive materials has been historically used to deliver a strong dose of radiation to a small, defined area of glioma tissue, while limiting the amount of radiation damage to nearby normal tissue. For instance, ^125^I seeds have been used for glioma and there is recent interest in the use of new forms of brachytherapy for glioma [31]. Two recent reviews detail findings [32,33] of brachytherapy in glioma patients. It was noted that there was clear survival advantage with postoperative radiation therapy at doses of 50 to 60 Gy, but dose escalation at doses higher than this resulted in increased toxicity without any survival benefit. For relapsed glioma, brachytherapy is also effective, but is associated with significant toxicity [32]. Here, we tested the systemic administration of small amounts (dosed at 100 µCi) of beta-emitting radionuclides delivered into the tumor microenvironment of GBM via a novel recombinant disintegrin called vicrostatin (VCN).

VCN was purposely designed with a carboxy-terminal extension, which was expected to enhance affinity for integrin α5β1, and this was confirmed as binding affinity of VCN for α5β1 was found to be ~13-fold higher than that of the native snake venom disintegrin, contortrostatin, VCN was modelled after. Mass spectrometry shows that VCN is predominantly a monomer with a MW of 7146 Da, a size which makes this molecule an ideal candidate for efficient penetration of the tumor microenvironment for payload delivery. Moreover, the high density of integrin targets expressed by the glioma neovasculature obviates the need for VCN to penetrate the blood-brain-barrier in order to deliver its ^131^I payload. According to the brachytherapy principle and provided that VCN sticks to the neovasculature, the beta particles emitted by ^131^I will have an effective energy release radius of about 2 mm into the tumor tissue. Regarding the ability of VCN to effectively ligate integrins, unlike cyclic RGD peptides and peptidomimetics, additional structural elements of VCN enable the disintegrin to engage the receptor in an efficient and unique manner which leads to rapid integrin internalization. Unlike cilengitide, cyclo(l-Arg-Gly-l-Asp-d-Phe-*N*-methyl-l-Val), a cyclic RGD peptide that was extensively tested in the clinic in GBM [34], the additional residues flanking the RGD-loop in VCN enable more extensive contacts with the integrin receptor. Also, NMR and crystallographic studies have revealed that the C-terminal tail in disintegrins folds in close proximity to the RGD-containing disintegrin loop, linking these two structural elements together in an extended conformational epitope, suggesting that these two functional regions are engaged in extensive interactions with the target integrin receptor [35,36,37]. This finding was further supported by our work on the rational design of VCN, accounting for a 13-fold improved affinity for α5β1, by incorporating changes to the amino acid sequence at the COOH-terminus of VCN [28]. By using structural and functional regions in addition to the RGD motif, a sequence that serves as the sole basis for the design of cyclic RGD peptides and RGD mimetics, disintegrins exhibit novel antitumor activities [27,28] compared to small cyclic RGD peptides and peptidomimetics. Due to its robustness and reproducibility, we believe that our recombinant production method will be easily translatable for scale up and cGMP production of VCN. Furthermore, toxicity studies in rodents using dose escalation of VCN (250 mg/kg to 500 mg/kg), a dose many times higher than that used in animal model studies of glioma, demonstrated complete lack of toxicity.

In the present study, we show that radioiodinated VCN can be used for precision delivery of therapeutic doses of ^131^I into the microenvironment of glioma. In an earlier biodistribution study, we demonstrated that radioiodinated VCN specifically binds to tumor tissue but not to the normal brain after intravenous administration [13]. Moreover, in two distinct animal models of glioma we show that radioiodinated VCN can significantly prolong the survival of treated animals. Specifically, in the TMZ-sensitive U251 xenograft model we show that ^131^I-VCN (100 μCi, intravenous) administered in combination with TMZ (25 mg/kg, oral gavage) more than doubles the survival of treated animals compared to the untreated controls. This result encouraged us to test the same therapeutic strategy in a syngeneic model of TMZ-resistant glioma. To establish this model, we transfected GL261 murine glioma cells with a plasmid that carries the murine *MGMT* gene under a mouse medium-strength promoter (i.e., the murine phosphoglycerate kinase 1 promoter). The transfected cells were further incubated in increased concentrations of TMZ (up to 700 μM) to select for highly TMZ-resistant clones which were further implanted in syngeneic hosts. In this model, we compared head-to-head the efficacy of ^131^I-VCN in combination with TMZ versus external beam radiotherapy in combination with TMZ. Although we expected all O6-methylguanine lesions to be directly reversed in the presence of murine MGMT, we reasoned that TMZ could still synergize with radiotherapy in this model via non-O6-methylguanine lesions, which in fact represent more than 90% of the DNA lesions inflicted by TMZ. Indeed, the combination of ^131^I-VCN plus TMZ proved superior to ^131^I-VCN alone which clearly suggest the possibility of a synergistic effect between TMZ and ^131^I-VCN in this setting. Moreover, two doses of ^131^I-VCN (100 μCi given twice) proved to be superior to five consecutive doses of whole-brain external beam radiotherapy (2 Gy given five times). These preliminary data suggest that, if dosed according to rigorous dosimetry studies, ^131^I-VCN may have the potential to significantly synergize with TMZ in the clinical setting of TMZ resistance (i.e., typically newly diagnosed patients with GBM tumors with unmethylated *MGMT* promoters). This could represent a therapeutic alternative to the current Stupp protocol [4] which only provides very limited benefit to this subset of patients.

We would like to make one additional point regarding the potential off-target effects of ^131^I-VCN in a clinical scenario. When compared to ^131^I sodium iodide which, even though it cannot discriminate between normal and cancerous thyroid tissue, is used in clinical practice for radioablation of cancerous thyroid cells, radioiodinated VCN is a much bigger molecule (i.e., a polypeptide of ~7 KDa). Due to its size, it is highly unlikely that VCN will fit into the Na-I symporter expressed by thyroid cells. For this reason, we predict that ^131^I-VCN will not accumulate in the thyroid glands of GBM patients treated with this form of intravenous brachytherapy. Moreover, the normal thyroid tissue is not known to express the integrin receptors expressed by the GBM tissue, which are ligated by VCN. However, because a potential off-target/off-tumor accumulation of radioactive material remains a concern with any form of radioiodine brachytherapy in clinical practice, we believe that any therapeutic protocol for ^131^I-VCN in glioma patients should include a pre-brachytherapy dosing step with potassium iodide. This preparatory step is intended to temporarily shut down the thyroid glands of GBM patients while ^131^I-VCN is present in the circulation.

In aggregate, our animal studies demonstrate that VCN can be successfully used as a novel delivery mechanism for targeting radioactivity to glioma cells. Although results are still preliminary, they serve as a proof-of-principle demonstration that radioiodinated VCN, which we tested in several animal models of GBM, can serve as an effective therapeutic approach for this deadly form of cancer. Further, the complexity of the cancer microenvironment dictates that for optimal efficacy an anti-integrin therapeutic must target at least two members of the RGD-binding integrin class and preferably more [38] given the ability of cancer cells to change their integrin repertoire in response to drug treatment. Our experience with VCN, which binds to multiple RGD-dependent integrins, suggests that it admirably meets this criterion while also showing minimal binding to normal tissue. Therefore, its excellent stability and high affinity for tumor integrins uniquely positions VCN for precision delivery of targeted radioactivity in GBM, a type of solid tumor where radiotherapy is an integral part of standard of care. In future investigations we will conduct dosimetry studies with VCN labeled with ^124^I (a positron emitter), which will allow us to precisely calculate by quantitative PET (positron emission tomography) imaging the therapeutic dose of ^131^I-VCN for each animal based on its tumor burden and integrin biology.

## 4. Materials and Methods

### 4.1. Cells and Reagents

The U87 and U251 human glioma cell lines were obtained from ATCC (Manassas, VA, USA) and maintained according to the manufacturer’s protocol. The GL261 murine glioma cell line was a gift from Dr. Alan Epstein (Keck School of Medicine, University of Southern California, Los Angeles, CA, USA). The TMZ-resistant variant of GL261 cell (i.e., GL261M) were generated by transfecting the wildtype cells with a plasmid construct carrying the mouse O6-methylguanine-DNA-methyltransferase (*MGMT*) gene under a mouse phosphoglycerate kinase 1 (mPGK) promoter. The transfected GL261 cells were further cultured in increasing concentrations of TMZ (up to 700 μM) to select the GL261M variant. VCN was produced and purified in the Markland laboratory [27,28]. Female 5-week-old 20 g Balb/c *nu/nu* mice were obtained from Simonsen Laboratories (Gilroy, CA, USA), and 5-week old C57 *bl/6* mice were obtained from Charles River Laboratories (Boston, MA, USA). Sodium iodine (^131^I) was obtained from Perkin-Elmer (Billerica, MA, USA). All other reagents were purchased from Sigma Chemical Co. (St. Louis, MO, USA). External beam radiotherapy was administered in a X-RAD320 irradiator (Precision X-Ray, North Branford, CT, USA). Temozolomide (TMZ) was obtained from TCI America (Portland, OR, USA).

### 4.2. Radioactive and Radioinert Iodination of VCN

VCN in solution was directly iodinated with radioiodine (^131^I) or radioinert sodium iodide using a modification of the chloramine T method [29]. Briefly, chloramine T was dissolved in PBS and added to a buffered VCN solution (100–600 µg at 0.3–2.0 mg/mL in PBS) containing Na ^131^I. Five successive aliquots of 15 µg chloramine T (in 5 µL) were added at 5-min intervals to the reaction mixture (final volume 400 µL). Following the final chloramine T addition, excess ^131^I was quenched through the addition of sodium metabisulfite (100 µg/400 µL reaction). Unreacted iodine was removed from the ^131^I-VCN solution and the buffer exchanged by 2× repeated filtration through a centrifugal 4-kDa cutoff filter (VWR, Atlanta, GA, USA). This allows iodine to pass through the membrane and be separated from the iodinated VCN solution.

### 4.3. Evaluation of Stoichiometry of Iodine Addition

The stoichiometry of iodine addition to VCN was determined by use of mass spectrometry. A LC-Electro-spray ionization mass spectrometry system was employed in the positive ion mode (Agilent, Santa Clara, CA, USA). A control of non-iodinated VCN was used as a standard.

### 4.4. FACS Analysis of VCN Binding to Glioma Cell Lines

The cold iodinated VCN (I-VCN) was further conjugated with Cy5-NHS (Lumiprobe, Hunt Valley, MD, USA) according to manufacturer’s protocol. For FACS analysis, 10^6^ glioma cells (U87, U251, and GL261M) in complete DMEM (Dulbecco’s Modified Eagle Medium) were incubated with or without 100-fold molar excess of a small cyclic RGD peptide called Cilengitide (Selleck Chemicals, Houston, TX, USA) [30] for 20 min at 37 °C, then pelleted and resuspended in ice-cold PBS. The cells were further incubated for 60 min at 4 °C with Cy5-I-VCN, then pelleted, washed and resuspended in ice-cold PBS. The cells were further analyzed on a BD FACSAria II instrument (BD Biosciences, Franklin Lakes, NJ, USA) equipped with a 642 nm red laser. Cells incubated with unlabeled disintegrin (i.e., negative control) were used to calibrate the instrument.

### 4.5. Confirmation of Biological Activity of Iodinated VCN

The inhibition of ADP-induced platelet aggregation by iodinated-VCN (both I-VCN and Cy5-I-VCN) was measured by determining the light absorption of human platelet-rich plasma (PRP) in a specialized spectrophotometer (Chrono-log 490 optical aggregometer, Chrono-log, Havertown, PA, USA) as previously described [39]. The iodinated disintegrins (I-VCN and Cy5-I-VCN) were tested for activity against unlabeled VCN.

### 4.6. Functional Analysis of GL261M Cells to Confirm MGMT Expression and Activity

Lysates prepared from wildtype GL261 and TMZ-resistant GL261M cells were analyzed by Western-blotting for MGMT expression levels. A rabbit polyclonal antibody that cross-reacts with the mouse MGMT protein was purchased from Boster Biological Technology (Pleasanton, CA, USA) and used in this Western-blot analysis. This antibody was raised against a synthetic peptide PVFQQESFTRQVLWK, which corresponds to a sequence in the middle region of human MGMT which is different from the related rat and mouse sequences by only one amino acid. Furthermore, to check the functionality of the MGMT enzyme resulting from transfection of the *MGMT* gene into GL261 cells, we completed a colony formation assay (CFA) with GL261M cells incubated in a range of TMZ concentrations in the presence or absence of the MGMT inhibitor O6-benzylguanine (Millipore Sigma, Burlington, MA, USA).

### 4.7. Establishment of Orthotopic Xenograft Glioma Models

All animal protocols were approved by the University of Southern California, IACUC (20707-CR001, 29 March 2018), and animals maintained according to approved guidelines. To perform this orthotopic xenograft model, tumors were implanted 3 mm deep in the midline of mice brains using stereotactic injections. Following surgical exposure of the skull and drilling a small-bore hole with a dental drill, U87 or U251 (2 × 10^5^/2 µL) glioma cells were injected using a slow controlled injection followed by a one-minute rest period with syringe in place to minimize leakage from the injection track. The syringe was slowly removed, the hole covered with wax, and the surgical incision was then closed with 2 stitches (3.0 silk). Following implantation (7–14 days) tumor take was evaluated by bioluminescent imaging. This was only possible in the U87 and U251 models in which the injected tumor cells were labeled with GFP and firefly luciferase. In these xenograft models, the tumors were imaged by intravenous injection of luciferin followed by a 90 s wait period when the animal was placed in a Xenogen IVIS 200 imaging instrument (Perkin Elmer, Waltham, MA, USA) before acquiring optical images. In our experience with these xenograft models, >90% of mice show a bioluminescent signal at 7 days post-implantation. Positive, tumor bearing, mice were then randomized into treatment and control groups.

### 4.8. Evaluation of Brain-Uptake of Radioiodinated-VCN

To determine tumor specific brain uptake and retention of iodinated VCN, mice were implanted with human gliomas (U87 as above) and the tumors were allowed to grow for 14 days. At this point mice with confirmed tumors and control non-tumor bearing mice were injected with a single dose of ^125^I-VCN (100 µCi) administered intravenously. Radioactively labeled VCN was allowed to circulate for 18 h at which time the animals were euthanized, and brains removed, rinsed and counted in a 2480 WIZARD2 gamma counter available in the Molecular Imaging Center at the Health Sciences Campus, University of Southern California.

### 4.9. Establishment of a Syngeneic Model of Glioma

For these studies, immunocompetent syngeneic C57BL/6 mice were implanted orthotopically with TMZ-resistant GL261M glioma cells. Following a protocol identical to the method used with the orthotopic xenograft model, tumors were implanted, and 13 days post implantation treatment was initiated. Due to immune rejection concerns, these tumor cells were not labeled with foreign transgenes such as GFP and luciferase, thus tumor growth could not be confirmed by bioluminescent imaging in this syngeneic model. However, based on survival curves that were previously generated by us after implanting various numbers of GL261M cells in vivo, we expected tumor growth in 100% of the mice implanted with 1 × 10^5^ GL261M cells.

### 4.10. Treatment of Animal Models

For the U87 glioma studies, the following groups were employed (five mice per group): (i) untreated control, (ii) treatment with ^131^I alone (administered IV as Na^131^I) and (iii) ^131^I-VCN. The dose of radioactivity administered was 100 µCi either iodine alone or radioiodinated VCN (^131^I-VCN). The mice were treated with a single dose and monitored daily for changes in weight and physical status. For the ^131^I-VCN combination with TMZ chemotherapy U251 glioma positive mice were randomized into 4 groups of 10 mice each. These groups were: (i) untreated control, (ii) ^131^I alone, (administered IV as Na^131^I), (iii) ^131^I-VCN alone, and (iv) ^131^I-VCN plus TMZ. The radioactivity was delivered as 100µCi either sodium iodine alone or radioiodinated VCN (^131^I-VCN) given in two doses separated by three days (each with 100 μCi ^131^I-VCN). The groups receiving TMZ had the agent delivered as a daily 25 mg/kg gavage over five consecutive days. The efficacy of ^131^I-VCN plus TMZ was further compared to external beam radiation therapy (EBRT) plus TMZ in a TMZ-resistant syngeneic model. In this model, we evaluated the following groups (five mice per group): (i) untreated control, (ii) ^131^I-VCN alone, (iii) EBRT plus TMZ and (iv) ^131^I-VCN plus TMZ. The radioactivity was delivered as 100 µCi either iodine alone or ^131^I-VCN given in two doses separated by three days (each with 100 μCi). In comparison, the EBRT plus TMZ group followed a dosing regimen mimicking the Stupp protocol [4] with TMZ delivered as a daily 25 mg/kg gavage concurrently with 2 Gy EBRT daily for five consecutive days. External beam irradiation was performed at 250 kv and 16 mA with a dose of 200 cGy delivered over 47 s to the skull of each animal over five consecutive days. Except for the skull area, the rest of each animal’s body was protected by a 3 mm-thick lead shield.

### 4.11. Statistical Significance

To determine statistical significance, the outcomes of the individual treatments were compared to the untreated controls using the Wilcoxon–Mann–Whitney two-sample rank-sum test. The treatment effect (difference between treatments) was quantified using the Hodges–Lehmann (HL) estimator, which is consistent with the Wilcoxon test. *p* values were determined and values less than 0.05 were considered significant.

## Figures and Tables

**Table 1:** Brain specific uptake of ^125^I-VCN.

Group	% of Injected Counts in the Brain
Control	No counts detected above background (200 cpm)
Tumor Bearing	1.4% of the injected counts
